# Influence of occlusal loading type and cyclic fatigue on the mechanical behavior of sound maxillary premolars: a laboratory and 3D finite element analysis

**DOI:** 10.1590/0103-644020256150

**Published:** 2025-08-11

**Authors:** Alexandre Coelho Machado, Paulo Vinícius Soares, Christian de Almeida Soares, Bruno Rodrigues Reis, Lívia Fávaro Zeola, Luís Henrique Araújo Raposo

**Affiliations:** 1 Department of Operative Dentistry and Dental Materials, Technical School of Health, Federal University of Uberlândia. Uberlândia- Minas Gerais, Brazil.; 2 Department Occlusion, Fixed Prosthodontics and Dental Materials, School of Dentistry, University of Uberlândia. Uberlândia - Minas Gerais, Brazil.; 3 Department of Restorative Dentistry, School of Dentistry, Federal University of Minas Gerais. Belo Horizonte- Minas Gerais, Brazil.

**Keywords:** Abfraction, cyclic fatigue, mechanical behavior, noncarious cervical lesion, stress-strain

## Abstract

This study evaluated the influence of different occlusal loading and cyclic fatigue on the biomechanical behavior of sound maxillary premolars using three-dimensional (3D) finite element analysis (FE) and strain-gauge tests. The 3D models were submitted to two occlusal loading (150 N): axial load (AL) and oblique load (OL), applied at the inner ridges. Dynamic fatigue was simulated in post-processing and analyzed by life criterion. For the experimental laboratory test, 30 sound premolars with similar dimensions had two strain gauges positioned parallel to the long axis of enamel and dentin to record the strains under AL and OL loading, before and after mechanical fatigue. FE maximum principal criteria showed higher stress concentration in the cervical enamel and dentin for OL (67.26 MPa and 17.43 MPa, respectively) compared to AL (0.87 MPa and 0.02 MPa, respectively). The equivalent elastic strain criterion showed higher strain values in dentin for OL (1.77e^-4^). The FE fatigue simulation showed higher damage for enamel near the cementoenamel junction with OL, predicting a lifespan of less than 200,000 cycles. Strain-gauge analysis showed that OL loading and mechanical fatigue resulted in higher strains, both enamel (p<0.001) and dentin (p<0.001). Very strong (0.927) and weak (0.184) correlations were found between the number of cycles and strain magnitude for enamel (p<0.0001) and dentin (p=0.0374), respectively. Non-axial occlusal loading, combined with cyclic mechanical fatigue, promotes high stress and strain concentration in the cervical enamel and dentin of sound premolars, potentially causing more damage to dental structures.

## Resumo

Este estudo avaliou a influência de diferentes cargas oclusais e fadiga cíclica no comportamento biomecânico de pré-molares superiores hígidos, utilizando análise tridimensional (3D) por elementos finitos (EF) e extensometria. Os modelos 3D foram submetidos a duas cargas oclusais (150 N): axial (AL) ou oblíqua (OL), aplicadas nas vertentes triturantes. Fadiga dinâmica foi simulada no pós-processamento e analisada pelo critério de vida útil. No ensaio laboratorial, 30 pré-molares hígidos com dimensões semelhantes receberam dois extensômetros posicionados paralelamente ao longo eixo no esmalte e na dentina para registrar as deformações sob as cargas AL e OL, antes e após a fadiga mecânica. O critério de tensão máxima principal demostrou maior concentração de tensão no esmalte e dentina cervical para OL (67,26 MPa e 17,43 MPa, respectivamente) comparado a AL (0,87 MPa e 0,02 MPa, respectivamente). O critério de deformação elástica equivalente demonstrou maiores valores de deformação na dentina para OL (1.77e-4). A simulação de fadiga por EF demonstrou maior dano para o esmalte próximo à junção cemento-esmalte com OL, prevendo vida útil inferior a 200.000 ciclos. A extensometria demonstrou que OL e fadiga mecânica resultaram em maiores deformações, tanto no esmalte (p<0,001) quanto na dentina (p<0,001). Correlações muito fortes (r=0,927) e fracas (r=0,184) foram encontradas entre o número de ciclos e a magnitude da deformação para o esmalte (p<0,0001) e dentina (p=0,0374), respectivamente. A carga oclusal não-axial, combinada com fadiga mecânica cíclica, promoveu alta concentração de tensões e maior deformação no esmalte e na dentina cervical dos pré-molares hígidos, causando potencialmente mais danos às estruturas dentais.

## Introduction

Wear of dental structure at the cementoenamel junction (CEJ) is an increasingly common finding in clinical practice, with reported mean prevalence rates of 47% and a frequency ranging from 9 to 93% in some populations ^1^. This structure loss is known as a noncarious cervical lesion (NCCL), and its multifactorial etiology is related to the association of mechanisms such as friction, bio corrosion, and stress ^2^. Friction is characterized as the wear of tooth substance caused by attrition and abrasion; while biocorrosion is the chemical, biochemical, and electrochemical degradation caused by endogenous and exogenous acids, proteolytic agents, and the piezoelectric effects on dentin ^2^; and lastly, stress is manifested as abfraction results caused by stress-strain concentration in dental structure originating from occlusal contacts (loading) ^2^.

The location and direction of occlusal loading can promote very distinct stress concentrations and strain patterns in the tooth structure ^3^ ^,^ ^4^. Higher stress concentrations are usually found in the cervical enamel and dentin when teeth are subjected to non-axial occlusal loading and/or parafunction ^3^ ^,^ ^4^ ^,^ ^5^. However, both functional and non-functional cyclic occlusal loads will determine the fatigue failure of the sound dental structure and may result in dormant cracks, contributing to the weakening of teeth ^4^ ^,^ ^6^ ^,^ ^7^. These cracks tend to initiate and grow according to the intensity of stress-strain and several occlusal cyclic loads ^6^ ^,^ ^7^, exceeding the enamel and dentin ultimate tensile and compressive strengths ^3^ ^,^ ^5^ ^,^ ^8^.

Due to the constriction in the cervical region of teeth, along with the structural micro-anatomy and composition of the dental hard tissues, this area is considered more susceptible to the etiologic factors of non-carious lesions ^4^ ^,^ ^9^. The reduced enamel thickness in this region and the perpendicular orientation of prisms ^10^ results in a less rigid ^11^, and more brittle dental structure when compared to occlusal enamel ^8^. The dentin at the cervical region is also more susceptible to failure ^8^ since the dentinal tubules are located transversely, in greater amounts, exhibiting large diameters ^10^ ^,^ ^12^.

The stress-strain pattern and fatigue processes occurring in teeth during function are complex mechanisms to be analyzed, and the association of methodologies for evaluating these variables is important ^3^ ^,^ ^4^. Understanding the stress distribution and strain values in various scenarios, as influenced by the direction and number of loading cycles, is essential in comprehending the various mechanisms underlying the formation of non-carious cervical lesions (NCCLs). Finite element analysis and strain-gauge tests are pivotal in providing insights into these phenomena. These methodologies offer valuable contributions in elucidating the influence of stress-strain patterns on the development of NCCLs ^3^ ^,^ ^4^ ^,4)^. Thus, the purpose of this study was to evaluate the influence of different occlusal loading types and cyclic mechanical fatigue on the stress-strain pattern distribution of sound maxillary premolars using a three-dimensional (3D) finite element analysis and strain-gauge test. The null hypothesis is that different occlusal loadings and cyclic mechanical fatigue do not impact the biomechanical behavior of sound maxillary premolars.

## Materials and methods

### Finite element (FE) analysis

**Figure 1:**
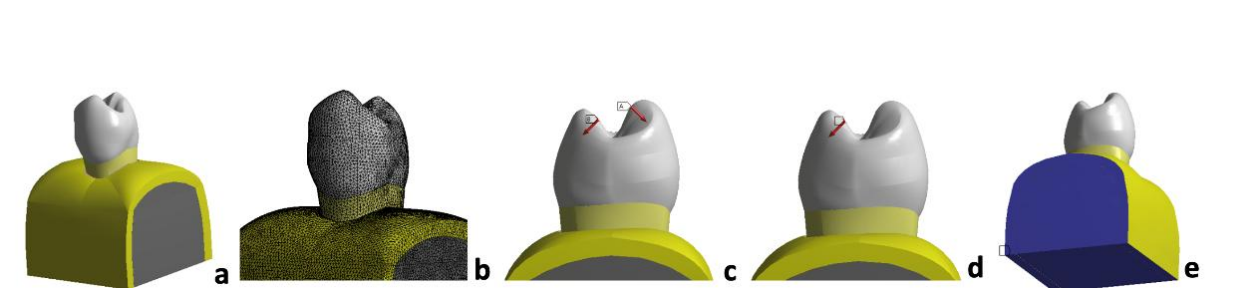
3D finite element models and boundary conditions. A- Volume of sound maxillary premolar; B- Meshing of the model; C- Axial loading (AL - 150 N); D- Oblique loading (OL - 150 N); E- Displacement restriction in X, Y, and Z axes (null displacement showed in blue).

A virtual linear 3D model of a single-rooted sound maxillary premolar was generated using anatomically-based geometric representations for enamel, dentin, pulp, periodontal ligament, cortical and trabecular bone ^3^. This model was exported in a STEP file (STP) to the FE analysis software (Ansys Workbench 17.0.1, Canonsburg, PA, EUA) (Figure 1A), and the mechanical properties were assigned to each structure as detailed in Table 1 ^11^ ^,^ ^13^ ^,^ ^14^ ^,^ ^15^. After a mesh conversion test, 10-node solid quadratic tetrahedral elements were selected for meshing (Figure 1B), totalizing, 1,709,931 elements with 2,473,693 nodes. The model was submitted to two occlusal loading schemes (150 N): axial load (AL) and oblique load (OL) (Figures 1C and 1D) ^3^ ^,^ ^4^. For AL, occlusal loading was applied in the inner ridges of both buccal and palatal cusps, at a 45-degree angle to the tooth-long axis. For OL, occlusal loading was applied only in the inner ridge of the palatal cusp, at a 45-degree angle to the tooth-long axis. The occlusal loading consisted of compressive pressure applied to the surface regions specified for each group (AL or OL). Models were restricted at the base and lateral surfaces of the cortical and trabecular bone in all translations (X, Y, and Z axes) (Figure 1E) ^3^. After processing the numerical simulations, results were plotted using maximum principal stress and equivalent elastic strain criteria, considering that failure of brittle structures is mainly attributed to the presence of tensile stresses. The highest and mean strain values were collected from enamel and dentin, considering all surface nodes in the areas where the sensors were attached during the strain gauge test, as described below.

**Table 1:** Mechanical properties assumed for orthotropic and isotropic structures ^11^ ^,^ ^13^ ^,^ ^14^ ^,^ ^15^. ^11^

Orthotropic Structures			
	Longitudinal	Transversal	Z
Elastic Modulus (MPa)			
Enamel	73,720	63,270	63,270
Dentin	17,070	5,610	5,610
Shear coefficient (MPa)			
Enamel	20,890	24,070	20,890
Dentin	1,700	6,000	1,700
Poisson Ratio (v)			
Enamel	0.23	0.45	0.23
Dentin	0.30	0.33	0.30
Isotropic Structures (13-15)			
	Elastic Modulus (MPa)		Poisson Ratio (v)
Pulp (13)	2.07		0.45
Periodontal Ligament (14)	68.9		0.45
Cortical Bone (15)	13,700		0.30
Medular Bone (15)	>1,370		0.30

For fatigue simulation using FE, the results for both types of static loads (AL and OL) were post-processed by using fatigue mechanic laws ^6^. The fatigue process was performed by calculating the number of cycles up to fatigue failure for multi-axial cyclic load. This approach was based on a linear elastic analysis and one-axial stress-life cycle (S-N) curves for enamel and dentin ^3^. For this, the stress-life cycle behavior and the apparent fatigue limit for enamel and dentin were calculated according to the following equation, σ_a_ = (σ_f_ - σ_m_) × (2N_f_)^b^, where σ_a_ is the stress failure; σ_f_ is the fatigue strength coefficient; σ_m_ is the mains stress; N_f_ is the number of cycles required for failure; and b is the fatigue strength exponent. In the present investigation, the main stress was assumed equal to zero, therefore, fatigue cycles were considered fully reversed (σ_m_ = 0). According to the enamel (σ_f_ = 310.0 MPa; b = -0.111) and dentin (σ_f_ = 247.0 MPa; b = -0.111) properties ^6^, the stress failure (σ_a_) was calculated considering 200,000 loading cycles and this data was assigned to the FE software. The ultimate tensile and compressive strength were 10.3 MPa and 384.0 MPa for enamel, and 98.7 MPa and 297 MPa for dentin, respectively ^16^. The FE fatigue simulation was plotted for enamel and dentin considering fatigue-life and fatigue-damage criteria ^6^ ^,^ ^17^.

### Strain-gauge method and mechanical fatigue simulation

For the strain-gauge test, 30 single-rooted sound human maxillary premolars free of clinical cracks and defects were selected (gathered following informed consent approved by the Ethics Committee on Research: #539.002). The specimens were embedded in polystyrene resin cylinders with periodontal ligament simulation. Two strain-gauges (PA-06-038AB-120LEN; Excel Sensors, São Paulo, SP, Brazil) were positioned in the cervical region parallel to the tooth’s long axis on the buccal surface, 2.0 mm above (enamel) and 2.0 mm below (dentin) the cement-enamel junction. In addition, two strain gauges were attached to a passive specimen mounted adjacent to the tested tooth to compensate for temperature and vibration fluctuations due to gauge electrical resistance or local environment ^7^.

**Figure 2:**
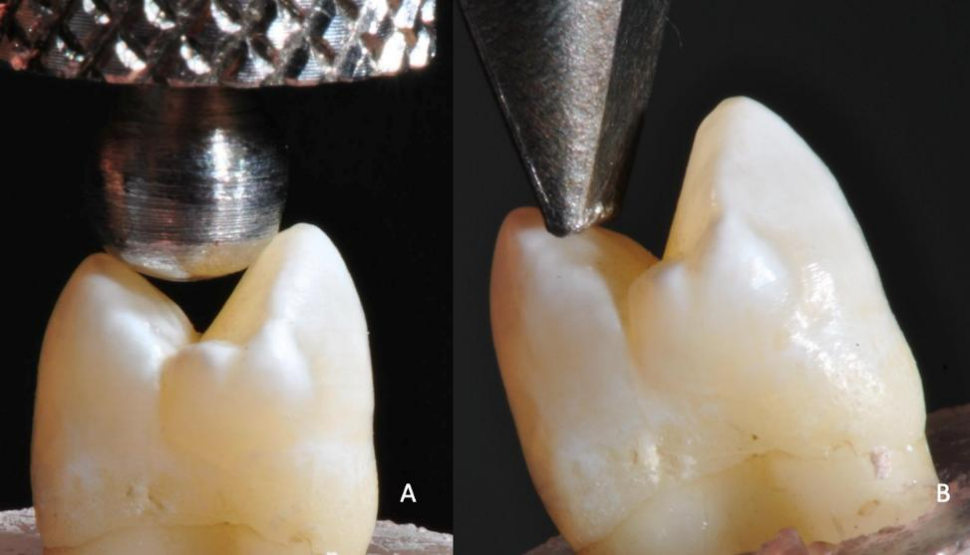
Sound maxillary premolars subjected to the different occlusal loading schemes: A- axial loading applied at the inner ridges of both cusps at a 0-degree angle to the tooth long axis (AL); B- oblique loading applied at the inner ridge of the palatal cusp at a 45-degree angle to the tooth long axis (OL).

Firstly, the specimens were subjected to a nondestructive compressive ramp-load from 0 to 150 N, at 0.5 mm/min in a mechanical testing machine (DL 2000; EMIC, São José dos Pinhais, PR, Brazil). The AL loading was applied at both cusps on the occlusal surface (inner ridges) using a 4.0 mm diameter sphere at a 0-degree angle to the tooth-long axis. For OL, a knife-shaped tip was used for loading the inner ridge of the palatal cusp, at a 45-degree angle to the tooth-long axis. Both AL and OL followed the same loading configuration used in the FE numeric simulations (Figure 2). At this step, the strains occurring at enamel and dentin were recorded by the strain gauges attached to the testing specimen. Sequentially, all specimens were immersed in distilled water at room temperature (25±2 ^o^C) and submitted to cyclic mechanical fatigue (200,000 cycles, 2 Hz, ER - 11000 Plus, Erios, São Paulo, SP, Brazil ) with non-axial oblique loading applied in the inner ridge of the palatal cusp (50 N), simulating approximately 10 months of clinical service ^18^ in a critical loading scenario ^3^.

After the fatigue protocol, new strain gauges were attached to the specimens at the same sites as previously, and the specimens were subjected to axial (AL) and oblique (OL) loading schemes for new strain measurements. The structural deformation data were collected from the strain gauges using a data acquisition device (ADS0500IP; Lynx, São Paulo, Brazil) combined with analysis software (AqDados 7.02 and AqAnalisys; Lynx, São Paulo, SP, Brazil). The strain data of enamel and dentin were checked for normality and homogeneity with Shapiro-Wilk and Levene’s tests, respectively. After confirming homoscedasticity, data were analyzed using two-way analysis of variance with repeated measures followed by Tukey’s HSD test. The Pearson correlation test was used to assess the relationship between the number of cycles and strain measurements (magnitude) performed for enamel and dentin under combined AL and OL loadings. All tests were performed at a 95% significance level (α = 0.05) using statistical software (SigmaPlot v. 12.2; Systat Software, Inc., San Jose, CA, USA).

## Results

### Finite element (FE) analysis

**Figure 3:**
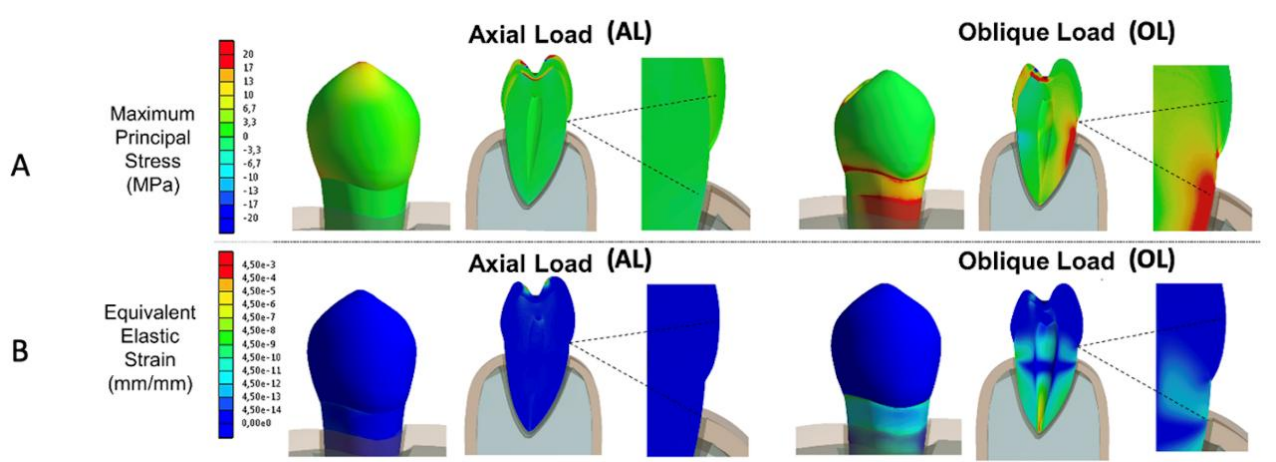
Results of finite element numeric simulations plotted according to the occlusal load type for: A- Maximum principal stress-strain pattern for enamel and dentin (positive and negative values indicate tensile and compressive stresses, respectively); B- equivalent elastic strain (regions from light blue to red colors represents higher deformation).

**Figure 4:**
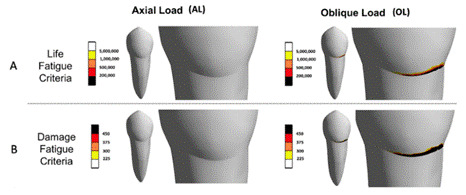
Results of finite element numeric simulations plotted according to the occlusal load type for: A- life fatigue criteria (areas with lower values from black to red colors tend to fail early); B- damage fatigue criteria (the red to black colors correspond to structures that received more damage during simulation).

The post-processing results of FE numeric simulations for the stress-strain pattern and fatigue criterion under the different loading conditions are shown in Figures 3 and 4, respectively. The oblique load (OL) loading presented a considerably higher stress concentration near the cementoenamel junction (CEJ) at the buccal surface of enamel (OL: 67.26 MPa; AL: 0.7MPa) and dentin (OL: 17.43 MPa; AL: 0.02 MPa), compared to axial load (AL) (Figure 3). In this case, the differences in stress magnitude between OL and AL occurring at the CEJ were increased 77 times for enamel and 871 times for dentin. Similarly to the stress distribution, lower strain values were observed for AL loading compared to OL in this region, regardless of the structure analyzed. For buccal CEJ enamel, the average strain value with OL was 1.4e^-4^, compared to 1.0e^-4^ with AL; and for dentin, the disparity between strains for OL and AL was about 2.9 times (OL: 8.7e^-4^; AL: 3.0e^-4^) (Figure 3). The buccal enamel at the cervical region showed high damage promoted by OL loading, and this area also presented the lowest values for structural fatigue-life criteria (with lower life prediction, withstanding less than 200,000 cycles) (Figure 4). Both FE fatigue criteria analyses showed a non-critical area near the buccal CEJ for the AL loading (Figures 3 and 4). However, this was not observed for the OL loading, which proved to be considerably more critical.

### Strain-gauge analysis

**Figure 5:**
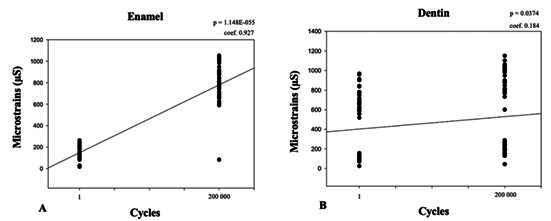
A- Pearson correlation test results plotted according to the number of mechanical cycles and strain magnitude (µS) assessed in enamel (A) and dentin (B), for both AL and OL loadings associated.

The mean strain values verified with the strain-gauge method for enamel and dentin at the buccal cervical region before and after mechanical fatigue are shown in Table 2. The deflection of the tooth structure was significantly higher for OL loading than for AL, both for enamel (p<0.001) and dentin (p<0.001), irrespective of mechanical fatigue. The specimens showed higher strains for both occlusal loading types after cyclic mechanical fatigue (OL: p<0.001; AL: p<0.001), irrespective of the dental structure evaluated (enamel or dentin). A very strong (0.927) and significant (p<0.0001) correlation was found between the number of mechanical cycles and the strain magnitude assessed in enamel. For dentin, a weak (0.184) but significant (p=0.0374) correlation was observed considering the number of mechanical cycles and the strain magnitude (Figure 5). These findings indicate that the greater the number of mechanical cycles, the higher the deformation experienced by the dental structures in the CEJ region.

**Table 2:** Mean strain values (µS) and standard deviation (±) for enamel and dentin according to occlusal load type and cyclic mechanical fatigue for each structure.

Enamel (µS)		
Load Type	Before Fatigue	After Fatigue
Axial Loading (AL)	90.10 ± 31.80 ^Aa^	135.88 ± 56.88 ^Ba^
Oblique Loading (OL)	179.05 ± 64.08 ^Ab^	208.82 ± 84.60 ^Bb^
Dentin (µS)		
Load Type	Before Fatigue	After Fatigue
Axial Loading (AL)	109.01 ± 17.31 ^Aa^	198.39 ± 30.59 ^Ba^
Oblique Loading (OL)	716.61 ± 83.28 ^Ab^	889.12 ± 86.38 ^Bb^

* Uppercase letters for horizontal comparisons (mechanical fatigue); Lowercase letters for vertical comparisons (load type); 2-way ANOVA-RM and Tukey HSD test (p<0.05).

## Discussion

Prior to the concepts of modern dentistry, the loss of enamel and dentin occurring near the cementoenamel junction (CEJ) had its etiology essentially based on isolated factors 2 ^) (^ 4 ^) (^ ^19^ ^) (^ ^19^ ^,^ ^20^ , ^(^ 2 ^,^ 4 , ^(^ ^19^. The exclusive friction caused by excessive toothbrush/dentifrice abrasion or the isolated biocorrosive degradation of dental tissues were initially considered the primary etiological factors for this localized loss of dental tissues ^19^ ^,^ ^20^. However, the presence of subgingival lesions, which would mainly be sheltered from frictional agents and partially protected from biocorrosive factors, or patients presenting only one tooth with cervical wear, raised new questions and furthered the theory of stress as an additional etiological factor ^19^. This concept, named abfraction theory, describes how stress concentration promoted by occlusal loads can disrupt the hydroxyapatite chemical bonds of enamel, and affect the crystalline structures of dentin ^2^. Small molecules, such as water or other elements, lodged between hydroxyapatite crystals can prevent the reestablishment of crystal bonds through remineralization, making this structure more susceptible to breakage and chemical dissolution ^4^ ^,^ ^21^. Thereafter, the term abfraction was used to describe the pathologic loss of enamel and dentin tissues due to stress concentrated away from the occlusal loading ^19^. Although labeling all NCCLs as a consequence of a single mechanism is inappropriate ^2^, the distinction between the terms “abfraction”, “corrosion” and “friction” is often misused and frequently applied to categorize all types of NCCLs ^2^ ^,^ ^5^.

The variation in the direction and intensity of occlusal loads is a relevant factor capable of causing changes of distinct magnitudes in the stress-strain pattern of the dental structure (3-4). In this study, the non-axial oblique occlusal load applied to the inner ridge of the (functional) palatal cusp resulted in higher tensile stress at the buccal cervical region in the numerical simulations with maxillary premolars (Figure 3). In situations where oblique occlusal loads are applied to the inner surface of the buccal cusps, higher compressive stress can develop and concentrate at the CEJ on the buccal side of the tooth ^3^. The origin and progression of NCCLs are mainly related to tensile rather than compressive stress due to the brittle behavior of enamel and dentin ^1^ ^,^ ^2^, which have lower ultimate tensile strength than compressive strength ^8^ ^,^ ^14^. Thus, according to our FE results and following previous evidence ^3^ ^,^ ^4^, the cyclic mechanical fatigue simulation was performed with a non-axial oblique load applied to the inner ridge of the palatal cusp in order to create a more critical scenario with harmful effects to the buccal cervical region (Figure 4).

Bite force may present considerable variations, fluctuating according to the measurement methodology, region (incisors, premolars, molars), sex, and conditions related to dental positioning and alignment ^22^ ^,^ ^23^ ^,^ ^24^. In the present study, the static force simulated through the FE method and strain gauge test was set at 150 N ^25^. This value corresponds to approximately 40% of the average forces recorded in the premolar region (366 N), considering both right and left sides, as well as male and female subjects ^22^ ^,^ ^23^. Some studies have reported higher values for this region during chewing (258 N) or swallowing (293 N), with the maximum bite force during clenching reaching up to 720 N ^24^. Although higher values could amplify the observed results, they would likely deviate from typical clinical scenarios.

As the different occlusal load types and mechanical fatigue impacted the biomechanical behavior of sound maxillary premolars in both numerical and laboratory analyses, the null hypothesis tested in this study was rejected. Failures of dental structures primarily occur in the presence of dynamic cyclic loads since the critical failure limit of tissues decreases compared to a single static load ^6^ ^,^ ^7^. This fact is justified, by the observation that cyclic loads result in stress fluctuations, and at a certain stage, can cause changes to the physicomechanical properties of dental tissues, resulting in the development and/or growth of dormant microcracks ^6^ ^,^ ^7^. Moreover, due to the multifactorial pathodynamic mechanism of NCCLs, the combination of friction and biocorrosion factors with the stresses resulting from occlusal loads can significantly exacerbate the degradation of the dental structure ^2^.

The high elastic modulus and low thickness of enamel at the CEJ are characteristics that favor stress concentration in this region (Figure 3) ^3^. Moreover, the presence of the Hunter-Schreger optical phenomenon may also facilitate stress concentration and crack propagation in the cervical region ^26^. This phenomenon occurs due to variations in the orientation of enamel rods in different layers of the tooth structure, creating alternating light and dark bands visible under reflected light ^27^. Since enamel is thinner and the dentin is closer to the surface in this region, variations in enamel rod orientation can lead to stress concentration and crack propagation ^27^ ^,^ ^28^. As a result, the different angles of the enamel rods can create weak points in this dental structure, making it more susceptible to failure under mechanical stress ^27^.

The main structures of interest in this study, enamel, and dentin, were considered orthotropic while assigning the mechanical properties for the numerical simulations using finite elements analysis ^7^ ^,^ ^11^. This is a significant aspect for more comprehensive FE investigations since these dental tissues can present distinct mechanical behavior depending on the tooth region and load direction ^11^. This difference is important due to the perpendicular disposition of enamel prisms and orientation of dentin tubules in the cervical region ^10^, in addition to the greater quantity and large diameter of these tubules ^12^. These aspects result in less rigid dental tissues, which are more susceptible to stress-driven failures in the cervical area than in other regions of the tooth ^8^. The FE fatigue-life simulation showed that the cervical enamel at the buccal side has a high probability of damage when the tooth is submitted to non-axial oblique loading in the inner ridge of the functional cusp (OL), withstanding minimal occlusal loads before initial fail occurs (Figure 4).

Also, for the strain-gauge test, the incidence of oblique loading for cyclic mechanical fatigue increased the strains at the cervical region both for enamel and dentin, regardless of the static load type (AL or OL). As seen, a very strong correlation was detected when relating the number of mechanical cycles and the strain magnitude assessed on the enamel surface. For dentin, a less marked but still significant correlation was also verified between these factors. This likely occurred because the structural strains were assessed on the cervical surfaces of enamel and dentin, and these outcomes are expected to reflect the strain distribution within a specific region of the tooth structure, rather than fully representing the internal strains of these tissues. Overall, these findings indicate a higher likelihood of damage occurring in the dental structures as a consequence of non-axial cyclic occlusal loads, even in a short period of time. For this reason, it is recommended to regularly examine patients predisposed to occlusal imbalances and NCCLs, and if necessary, perform occlusal equilibration to remove possible interferences ^3^ ^,^ ^29^. 

In addition to the occlusal aspects, identifying frictional habits and biocorrosive factors, even as slight clinical signals and symptoms, would contribute to reducing the onset and progression of NCCLs (2, 4, 20). There is strong evidence of an association between NCCLs and occlusal surface wear, with prevalence rates reaching up to 80.28% ^30^. Moreover, bruxism and clenching may be related to occlusal instability, also increasing the duration of teeth intercuspation with substantial loading and cervical stress ^4^ ^,^ ^5^ ^,^ ^31^. Constant occlusal contact from non-physiological loads may cause hyperemia and inflammation of pulpal tissues, causing increased sensibility ^32^. Furthermore, cervical dentin hypersensitivity is one of the first symptoms of NCCLs and it is prevalent in 70% of the teeth with NCCLs [3, 4]. These symptoms can also occur due to cervical enamel loss and exposure to dentin tubules, resulting from the NCCLs’ etiological factors ^2^.

Nondestructive research methodologies such as FE simulations and strain-gauge tests, are useful to assess the biomechanical behavior of specific clinical situations in dentistry ^3^ ^,^ ^4^ ^,^ ^6^ ^,^ ^27^. Although these methodologies allow the evaluation of different factors in the same specimens, limitations like homogenous FE models and extra-oral environment analysis may impact the results ^3^. Due to these limitations, an association of methodologies is relevant for validating the research results. In the present investigation, the influence of occlusal load types and cyclic mechanical fatigue was consistent with the results observed for enamel and dentin in both numerical FE simulations and laboratory strain-gauge tests. Further studies should focus on clinically investigating the various scenarios involving different aging conditions and occlusal loading patterns in relation to the formation of NCCLs.

## Conclusion

Within the limitations of this study, it can be concluded that the presence of non-axial (oblique) occlusal loads combined with cyclic mechanical fatigue promotes high stress and strain concentration, that may be more harmful to the dental structure of sound maxillary premolars at the cervical region (CEJ). Axial loads are less harmful and can be well tolerated by dental mineralized tissues, even in premolars that have been subjected to more critical fatigue scenarios. Thus, an occlusal loading pattern with primarily vertical resultant forces can help to reduce undesirable stress and strain concentrations in the CEJ region of posterior teeth.
